# Reversible Left Ventricular Systolic Dysfunction Secondary to Pazopanib

**DOI:** 10.7759/cureus.3517

**Published:** 2018-10-29

**Authors:** Kushani Gajjar, Kathir Balakumaran, Agnes S Kim

**Affiliations:** 1 Internal Medicine, University of Connecticut Health Center, Farmington, USA; 2 Cardiology, University of Connecticut Health Center, Farmington, USA

**Keywords:** cardiomyopathy, pazopanib, cardio-oncology, oncology, cardiotoxicity, tyrosine kinase inhibitors, vascular-endothelial growth factor

## Abstract

Pazopanib is a tyrosine kinase inhibitor used for the treatment of advanced renal cell carcinoma and advanced soft tissue sarcoma. We describe a case report of a patient with spindle cell sarcoma who developed severe left ventricular systolic dysfunction after starting pazopanib therapy with subsequent recovery of left ventricular ejection fraction upon stopping therapy.

## Introduction

Pazopanib is a vascular endothelial growth factor and tyrosine kinase inhibitor (VEGF-TKI) used in the treatment of advanced renal cell carcinoma (RCC) and advanced soft tissue sarcoma. They are known to cause adverse cardiovascular effects including hypertension, cardiomyopathy, thromboembolism and myocardial ischemia. We describe a case report of a patient with spindle cell sarcoma who developed severe left ventricular systolic dysfunction after starting Pazopanib therapy with subsequent recovery of left ventricular ejection fraction (LVEF) upon stopping therapy.

## Case presentation

**Figure 1:**
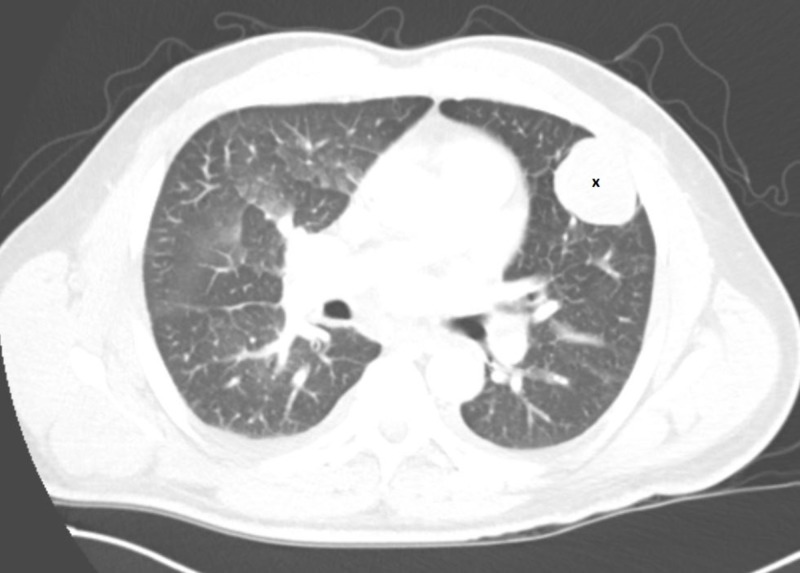
Computed tomography imaging of the lungs revealing large 4.5 cm x 5 cm circumferential pleural-based mass (x) consistent with metastatic sarcoma.

A 49-year-old man with a history of spindle cell sarcoma status post left arm below-elbow amputation developed recurrence of the sarcoma three years post amputation. He was found to have metastasis of cancer to the lungs on staging (Figure 1) and received three cycles of Doxorubicin and Olaratumab followed by wide excision of the soft tissue tumor.

**Figure 2:**
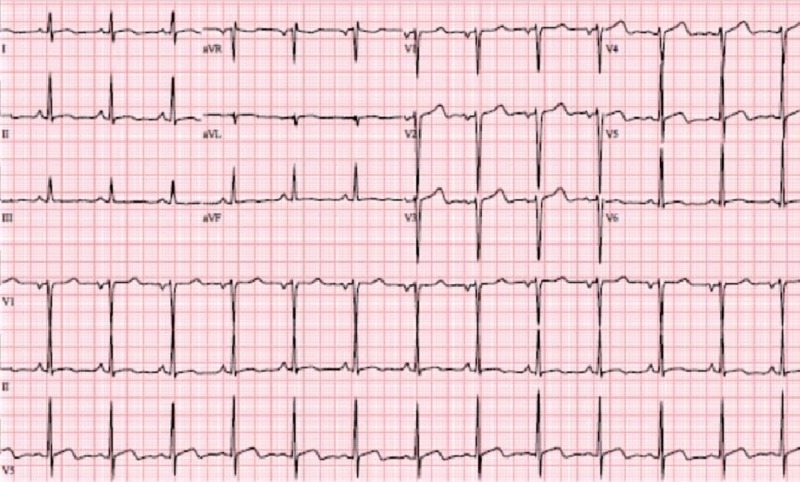
Admission electrocardiogram.

He underwent a transthoracic echocardiogram (TTE) six months after completion of Doxorubicin therapy, and his LVEF was found to be normal at that time. Unfortunately, he continued to have progression of pulmonary metastasis and hence was initiated on Pazopanib, which is a second line therapy for advanced soft tissue sarcomas. Shortly after initiation of therapy, the patient started developing palpitations, shortness of breath on exertion and chest tightness. His symptoms progressively worsened, until he suffered a syncopal episode 10 days after initiation of Pazopanib. On admission to the hospital, the patient’s physical examination was unremarkable. The laboratory data was remarkable for an elevated troponin I to 0.09 ng/dl on admission. His electrocardiogram (ECG) revealed nonspecific ST segment changes (Figure 2).

**Figure 3:**
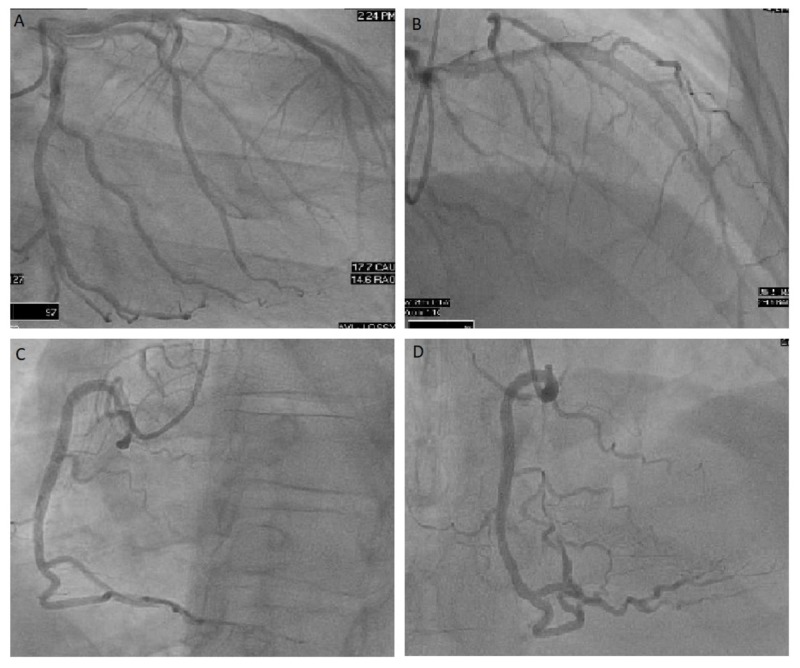
Coronary angiography. (A) Left anterior oblique view of revealing patent left coronary vasculature. (B) Right anterior oblique cranial view revealing patent left coronary vasculature. (C) Right anterior oblique view revealing patent right coronary vasculature. (D) Right anterior oblique view revealing patent right coronary vasculature.

As part of the workup for his syncope, he underwent a TTE, which revealed an LVEF of 10% to 15% with severe diffuse hypokinesis, right ventricular systolic dysfunction with normal biventricular chamber sizes. He subsequently underwent cardiac catheterization which revealed normal coronary anatomy (Figure 3).

He was initiated on guideline directed medical therapy with a beta blocker and angiotensin converting enzyme inhibitor and discharged with cardiology and oncology follow-up. His Pazopanib was stopped on discharge. The patient subsequently underwent repeat TTE six weeks from discharge, which revealed recovery of LVEF to 40 to 45% with normal cavity size and mild diffuse hypokinesis. A repeat TTE three months afterwards revealed an LVEF 45 to 50%. He was subsequently started on immunotherapy with Ipilimumab and Nivolumab combination therapy.

## Discussion

In RCC trials, myocardial systolic dysfunction with Pazopanib has been reported to be about 13% [1]. A meta-analysis of congestive heart failure (CHF) with VEGF-TKI shows a relative risk of all grade and high-grade CHF for the VEGF-TKI vs. no VEGF-TKI arms was 2.69 and 1.65, respectively [2]. In 362 Pazopanib-treated patients, a 1% incidence of symptomatic heart failure (HF) and 9% incidence of an absolute left ventricular ejection fraction decline of 15% or greater was observed [3]. Higher rates were described in a meta-analysis that included three trials (n = 314) and found an HF incidence rate of 6.1% [4]. There have been few case reports of heart failure induced by Pazopanib including apical ballooning and rapid fatal cardiac decompensation [5-8].

Pazopanib has a multimodal mechanism of action, including inhibiting cell surface VEGF and platelet-derived growth factor (PDGF) receptors. VEGF plays a central role both in maintaining a well-vascularized myocardium and in developing a robust neovascular response to chronic ischemic changes [9]. In a rabbit-model of left ventricular hypertrophy, promoting capillary growth with VEGF reduces apoptosis, preserves myocardial contractile function, and delays the onset of heart failure [10]. Moreover, PDGF expressed in cardiomyocytes, also a target of Pazopanib, has been demonstrated to exert a cardioprotective angiogenic function [11]. This thus suggests that VEGF and PDGF receptor inhibition could induce cardiomyocyte apoptosis and prevent cardiac remodeling, resulting in ventricular dysfunction. A recent study in 2017 suggests that the cellular targets likely to be involved in cardiac failure with protein kinase inhibitors may be ABL1 and ABL2 tyrosine kinases (Abelson Murine Leukemia Viral Oncogene Homolog) [12]. There is some evidence to suggest that TKI interruption along with optimal guideline-directed cardiovascular treatment leads to improvement in cardiac status and such patients can be eligible to resume TKI therapy [11].

Additionally, cardiotoxicity and subsequent left ventricular systolic dysfunction is a known complication of anthracyclines, however most cardiotoxicity after anthracycline-containing therapy occurs within the first year and is associated with anthracycline dose and LVEF at the end of treatment [13]. Our patient had normal LVEF six months after completion of anthracycline therapy and hence we believe that his acute heart failure was in the setting of VEGF-TKI administration. He also subsequently recovered his LVEF upon stopping the VEGF-TKI. The PALETTE trial showed a 1% rate of symptomatic left ventricular systolic dysfunction in people treated with Pazopanib, while 99% of patients had received anthracyclines [1]. This again supports the hypothesis that Pazopanib causes cardiotoxicity independent of prior exposure to anthracyclines.

## Conclusions

We believe this case report will add to the available literature about this potentially life-threatening complication associated with VEGF-TKI therapy while demonstrating the potential reversibility of cardiotoxicity with cessation of therapy. We summarize that close monitoring of cardiac function on Pazopanib and other VEGF-TKI inhibitors is necessary. These patients need close follow-up in specialized cardiology-oncology clinics and monitoring echocardiograms while on these medications. With improving understanding of the genetic variations involved in adverse cardiac events especially heart failure, it might be possible to use this knowledge to screen which patients are at a higher risk for these complications.
